# The Ethanol Supernatant Extracts of Liushenwan Could Alleviate Nanodiethylnitrosamine-Induced Liver Cancer in Mice

**DOI:** 10.1155/2018/6934809

**Published:** 2018-09-26

**Authors:** Xi-Zhen Chen, Wei Kevin Zhang, He-Bin Tang, Xiao-Jun Li, Gui-Hua Tian, Hong-Cai Shang, Yu-Sang Li

**Affiliations:** ^1^Department of Pharmacology, School of Pharmaceutical Sciences, South-Central University for Nationalities, Wuhan, China; ^2^Key Laboratory of Chinese Internal Medicine of MOE, Beijing Dongzhimen Hospital, Beijing University of Chinese Medicine, Beijing, China

## Abstract

Liver cancer is one of the leading causes of cancerous deaths worldwide. At present, the treatment of hepatocellular carcinoma (HCC) remains to be a problem globally. Liushenwan (LSW), an ancient Chinese medicine previously used to treat localized infections, was recently reported to possess anticancer activity. Here in this study, we aim to examine the effect of LSW-ET (LSW-ET is the supernatant fraction of LSW from ultrasound assisted ethanol extraction) in prevention and treatment on nanodiethylnitrosamine- (nanoDEN-) induced HCC in mice. In nanoDEN-induced HCC mice treated with LSW-ET by oral (po) or intragastric gavage (ig), we observed an alleviation of serum ALT and AST levels, amelioration in histopathological stainings, and an inhibition in liver tumor growth. In addition, compared with the nanoDEN group, downregulation of multiple pivotal factors (COX-2, *β*-catenin, PCNA, and HMGB-1) was observed in LSW-ET-po and LSW-ET-ig groups. Taken together, the delivery of LSW-ET by oral could be a potential prevention and treatment of liver cancer.

## 1. Introduction

Liver cancer, also called hepatocellular carcinoma (HCC), has been and would remain to be one of the leading causes of cancerous deaths worldwide nowadays and in the near future [1]. In China particular, liver diseases affect approximately 300 million people, which contributes much to the global burden of liver diseases [2]. A large fraction of these patients would probably progress into liver cancer at advanced stages due to late diagnoses [3]. In addition, large variability of treatment choices and disease-relate survival also confirmed such heterogeneity. However, a so-called “best” choice for patients with HCC could be quite hard [4]. Thus, alternative agents with better efficacy and less detriment for the management (prevention and treatment included) of HCC are urgently needed [5].

Traditional Chinese Medicine has been widely used for anticancer treatment in China with a long history and many substances possess application prospect, such as bufadienolide [6, 7]. Liushenwan (LSW), a well-known heat clearing and detoxifying herbal preparation, is composed of Niu-huang (Calculus bovis), She-xiang (Moschus), Chan-shu (Bufonis venenum), Xiong-huang (Realgar), Zhen-zhu (Margarita), and Bing-pian (Camphora). This ancient prescription is quite effective in treating localized infections and inflammation associated pain [8]. During the past 200 years, many other applications of LSW have been uncovered, including the treatment of diphtheria, scarlet fever, pharyngotonsillitis, acute tonsillitis, purulent parotitis, encephalitis B, and leukemia [9, 10]. Recently, it was also reported that LSW had a potential anticancer activity [9]. In the meantime, LSW has also been suggested to be associated with some toxicity due to the constituents of Realgar and Bufonis venenum, both of which were found to be hazardous once administered alone [11]. In our previous study, we demonstrated that the ethanol fraction of LSW (LSW-ET), which maintained its analgesic and anticancer activities, possessed a minimum side effect to a normal human hepatic cell line L02, while inhibiting cell proliferation and inducing apoptosis of the human cancerous cell line HepG2 in a dose-dependent manner via the downregulation of NK-1 receptor [12]. However, experiments on cancerous animal models were required to investigate the efficacy of LSW-ET in vivo.

It would be quite complicated for the occurrence and development of liver cancer, which could be separated into multiple stages and involved key regulatory factors and signaling pathways [13–16]. Abnormal expressions of canonical Wnt/*β*-catenin signaling pathway was closely related to malignancies, and its role in liver biology had come to the forefront over the last several years [17]. Under hepatic pathological conditions, an aberrant activation of *β*-catenin was evident in chronic inflammation, steatosis, fibrosis, and subsequently tumors of the liver [18, 19]. Another “active player”, COX-2, a key protein involved in the inflammation process [20], had also been suggested to play important roles in the relationship between inflammation and cancer. Studies had shown that the expression of COX-2 in para-carcinoma tissues was higher than that of tumor tissues and was also positively correlated with the degree of differentiation for liver tumor, suggesting a promoting role in the carcinogenesis of early stage [21]. Meanwhile, PCNA (a protein recently related with tumor formation [22]) and HMGB-1 (acts extracellularly as a cytokine to promote cell migration [23]) had been shown to be associated with liver cancer as well.

Therefore, in this study, to further evaluate the efficacy and detriment of LSW-ET in vivo, we employed a nanoDEN-induced liver cancer model of mice previously introduced by us [24] and examined the tumor growth rate, hepatotoxicity, and histopathological changes after the treatment of LSW-ET. Furthermore, we investigated expression changes of several pivotal factors during the progression of HCC via histochemical stainings, including *β*-catenin, COX-2, PCNA, and HMGB-1.

## 2. Materials and Methods

### 2.1. Materials

LSW was purchased from shanghai ley's pharmaceutical Co., Ltd. (Shanghai, China). Diethylnitrosamine (DEN) was purchased from Tokyo Chemical Industry Co., Ltd. (Tokyo, Japan). Tween-80 was kindly provided by Amresco®. (Amresco, USA). Lecithin from egg yolk was purchased from Sinopharm Chemical Reagent Co., Ltd. (Shanghai, China). Sesame oil was kindly provided by Blessing Mill (Wuhan, China). Glycerol ReagentPlus (Gas chromatography [GC] grade) were obtained from Sigma-Aldrich Co. (St Louis, MO, USA). Ultrapure deionized water (Heal Force Bio-Meditech Holdings Ltd., Shanghai, China) was used for all the experiments. All other reagents were of analytical grade. Primary antibodies used in this study included antibodies against *β*-catenin, PCNA, HMGB-1, and COX-2 (Abcam Inc., Cambridge, MA, USA).

### 2.2. HPLC Analysis of LSW-ET

The chemical components of LSW-ET were detected by HPLC DionexTM Ultimate 3000 system (Thermo Fischer Scientific, Inc., USA) equipped with an Ultimate 3000 pump, an Ultimate 3000 autosampler, and an Ultimate 3000 column compartment. Separation was performed using a C18 column (4.6 × 250 mm, 5 *μ*m, Kromasil) and an optimized mobile phase composed of acetonitrile (solvent A) and 0.2% formic acid water solution (solvent B, v/v). For the combinative elution, elution was achieved using the following conditions: 0-3 min, 9% solvent A; 3-13 min, 9-12% solvent A; 13-15 min, 12-20% solvent A; 15-25 min, 20-30% solvent A; 25-30 min, 30-51% solvent A; 30-50 min, 51% solvent A (total run time 50 min). The detection wavelength was set at 296 nm and the flow rate was 0.8 mL/min. The column temperature was set at 30°C and the injection volume was 10 *μ*L.

### 2.3. Animal Care

Male Kunming mice (18–22 g) were acclimatized for seven days under SPF conditions before the initiation of experiments. The animals were kept in a temperature controlled laboratory (22–25°C) with a 12-hour light–dark cycle. The care and use of animals and experimental protocols for this study were performed according to the Committee of Research Facilities for Laboratory Animal Sciences. All studies on mice were approved by the Committee on the Ethics of Animal Experiments of the South-Central University for Nationalities, Wuhan, China (permit number: 2012-SCUEC-AEC-005).

### 2.4. Evaluation of the Tumor Growth Inhibition

A liver cancer model induced by nanoDEN with modifications was employed according to a previous study [24]. In brief, 128 male Kunming mice were randomly divided into four groups: the control, model, LSW-ET-ig, and LSW-ET-po groups. All groups were orally given nanoDEN (16.5 mg / kg) once a week for twenty consecutive weeks, except for the control group which was applied with vehicle treatment. Meanwhile, LSW-ET-ig or LSW-ET-po (9.639 mg/kg in sesame oil) group was treated with LSW-ET intragastrically or orally respectively, twice a week for thirty weeks. Body weights were measured daily until the mice were sacrificed at week 10, 15, 20, and 30 (the day of the first administration of LSW-ET and nanoDEN was week 0). Liver samples from all mice were collected. While a part of each liver was used for histopathological analysis, the rest of the liver was stored at −80°C fridge.

### 2.5. Liver Blood Tests of Mice

Blood (1 ml) for each mouse was collected at sacrifice and centrifuged at 1000 rpm for 5 min. Serum was stored at −20°C for serum chemistry profile analysis. The levels of alanine aminotransferase (ALT), aspartate aminotransferase (AST), total bilirubin (TBIL), alkaline phosphatase (ALP), triglycerides (TRIG), and low-density lipoprotein-cholesterol (LDL-C) were determined using DRI-CHEM 7000i automated clinical chemistry analyzer (Fujifilm Corporation, Tokyo, Japan).

### 2.6. Histopathological Analysis of Liver Tissues

Fresh liver tissue pieces were fixed in 10% formalin, dehydrated, paraffin embedded successively, sliced at 2 *μ*m, and subsequently followed by sectioning and hematoxylin and eosin (H&E) by standard techniques. Histopathologic examinations of the liver sections were conducted using a Nikon 50i light microscope (Nikon Inc., Tokyo, Japan) by a pathologist and peer-reviewed.

### 2.7. Immunohistochemical of Liver Tissues

Samples were deparaffinized, hydrated, and blocked with 3% hydrogen peroxide for 15 min. Then specimens were subjected to antigen retrieval by immersing in 0.01 M boiling citrate buffer and heating in a microwave oven for 10 min. After blocking by 5% BSA (Boster, Wuhan, China) for 1 hour, the sections were incubated overnight at 4°C with primary antibodies against *β*-catenin (1:200), COX-2 (1:200), PCNA (1:6000), and HMGB-1 (1:1000), respectively. After incubation with horseradish peroxidase- (HRP-) conjugated secondary antibodies (Boster, Wuhan, China) at 37°C for 1 hour, the expression was visualized by 3,3′-diaminobenzidine tetrahydrochloride (Boster, Wuhan, China) staining. Hematoxylin was used as a nuclear counterstain in tissue sections. Stained slides were dehydrated by sequential steps through a graded series of alcohol washes and mounted using coverslips. Multispectral images was obtained using the Nuance Multispectral Imaging System (Cambridge Research and Instrumentation Inc., Woburn, MA) as described in our previous study [25]. Briefly, spectral optical density data were automatically acquired from 420 to 720 nm in 10 nm increments. Spectral unmixing was accomplished by the Nuance software (v3.0.2) and pure spectral libraries of individual chromogens. Nonspecific background staining was subtracted from each image individually. For the quantification in each experiment, three equal-sized fields of each photograph per group were randomly chosen.

### 2.8. Statistical Analysis

All results were presented as mean ± SEM. Statistical analysis was carried out by a one-way analysis of variance (ANOVA, followed by Bonferroni post hoc tests if necessary) in Origin8.0 (Origin lab Corp., MA, USA).* P *< 0.05 was considered to be statistically significant, and the levels of differences were indicated in the legend of each figure as follows: compared with the normal group, *∗P *< 0.05, *∗∗P *< 0.01, *∗∗∗P *< 0.001, and †*P *> 0.05; compared with the nanoDEN group, #*P *< 0.05, ##*P *< 0.01, ###*P *< 0.001, and ‡*P *> 0.05.

## 3. Results

### 3.1. Component Analysis of LSW-ET

**Figure 1:**
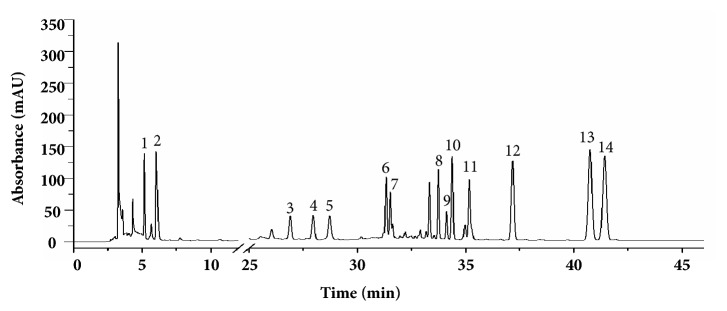
Separation of the alcohol-soluble extractants of LSW by using HPLC.

As shown in Figure 1 and Table 1, with the help of LC-MS and reference substances, telocinobufagin, bufotaline, cinobufotalin, bufalin, cinobufagin, and resibufogenin had been identified as main components of LSW-ET, most of which have been reported to possess anti-inflammatory, antitumor, and cardiotonic effects [26–28].

### 3.2. Oral Uptake of LSW-ET Phenotypically Relieved Liver Damage in Mice

**Figure 2:**
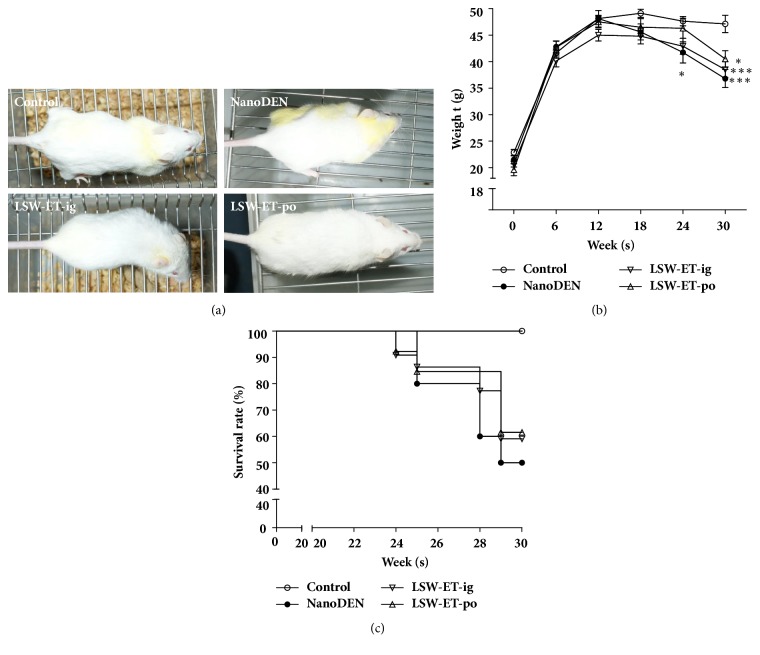
**The overviews of mice with different treatments. **(a) General view of the mice. (b) Gain of body weight in different groups during the experiment. (c) Survival rates of mice in various groups during the liver carcinogenesis. *∗* and *∗∗∗*,* P *< 0.05 and 0.001, respectively; †,* P *> 0.01 compared with the control group; ‡,* P *> 0.01 compared with the nanoDEN group.

To explore the effect of LSW-ET on liver cancer in vivo, a model induced by nanoDEN was employed. In our experiments, we observed a severe damage in livers of mice after the application of nanoDEN as shown in Figure 2. Phenotypically, mice in each group were almost identical with others in the first 15 weeks. By careful measurement of their weight, mice with all treatments were identical to each other, suggesting a relative equal intake of food and water among mice in different groups. However, at the 20th week, the hair of mice in the nanoDEN group appeared to be dull and yellowish, while they were also thinner and lighter compared with other treatment groups (Figure 2(a)). The differences of body weight could be due to the occurrence of liver tumor, which affected the appetite of these mice.

Moreover, significant toxicity was observed in the nanoDEN group by weight loss while LSW-ET-ig and LSW-ET-po treatments relieved a bit (Figures 2(b) and 2(c)). As shown in Table 2, the levels of alanine transaminase (ALT), aspartate transaminase (AST), and alkaline phosphatase (ALP) were significantly elevated in the nanoDEN group suggesting that nanoDEN, as demonstrated previously, could cause the hematologic disorders. In comparison, treatments of LSW-ET-po and LSW-ET-ig could alleviate this nanoDEN-induced liver cytotoxicity. It would also be interesting to note that LDL-C level was significantly decreased in nanoDEN-treated group but recovered after both treatments of LSW-ET-ig and LSW-ET-po.

**Figure 3:**
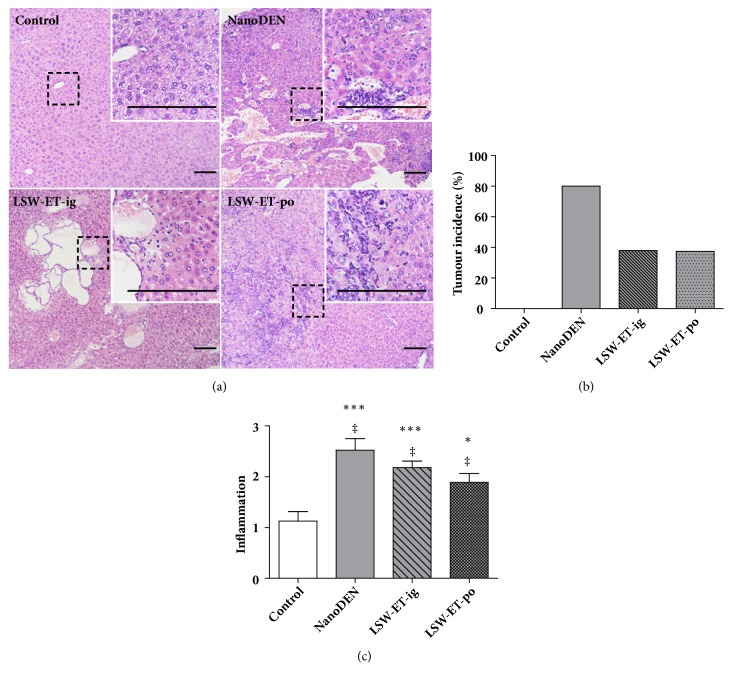
**The anticancer efficacy of different administration of LSW-ET in nanoDEN-induced HCC in mice. **(a) Histological analysis of livers from the control group, nanoDEN group, LSW-ET-ig, and LSW-ET-po group at the 30th week. Histological analysis of the livers was performed by hematoxylin and eosin staining. Part of the arbor (stippled outline, 100×) is shown enlarged in the inset (400×) in the top right corner. Scale bar, 50 *μ*m. (b) The probability of occurrence of tumor in each group. (c) Scores of inflammation of liver samples. *∗* and *∗∗∗*,* P *< 0.05 and 0.001, respectively, compared with the control group; ‡,* P *> 0.01 compared with the nanoDEN group.

Liver specimens of mice in different treatment groups were harvested at weeks 10, 15, 20, and 30. In Figure 3(a), we could observe from the representative histopathological stainings in the week 30 mice that there were severe inflammatory infiltration and weakly extensive collagen deposition and pseudo-lobular formation in nanoDEN group, whereas liver cancer (80%; 4/5) occurred frequently with an increased nuclear-to-cytoplasmic index, enlarged and hyperchromatic nuclei, and expansive growth. In the contrary, intragastric injection of LSW-ET decreased the development of tumor nodules, but their inflammatory infiltration and hepatocyte necrosis were noted in the adjacent tissues. However, oral treatment of LSW-ET decreased the development of tumor nodules. In the meantime, normal architecture of the adjacent tissues remains intact.

These results indicated an in vivo antitumor effect of ethanol extracts of LSW, especially after oral treatment. To further quantify the difference among different treatments, scores of inflammation were introduced as reported previously [29]. The results showed that the inflammatory infiltration (inflammatory score: 2.52 ± 0.23) in the nanoDEN group was significantly increased at the thirtieth week compared with the control group, and the degree of inflammation in the liver tissues of mice treated with LSW-ET group (LSW-ET-ig group: 2.18 ± 0.13; LSW-ET-po group: 1.89 ± 0.18) was decreased, suggesting an anti-inflammatory effect of LSW-ET on liver. The overall scores of inflammation and incidence of tumor further strengthen the idea that ethanol extracts of LSW could alleviate nanoDEN-induced cancer (Figures 3(b) and 3(c)).

### 3.3. Expressions of Inflammatory and Tumor-Related Proteins Were Decreased after LSW-ET Treatment In Vivo

To further demonstrate the efficacy and efficiency of LSW-ET, we monitored changes of several pivotal factors, which had already been demonstrated to be greatly increased in liver samples of nanoDEN-treated mice [18, 24]. As shown in the following figures, expressions of all these four factors were elevated as well in the nanoDEN-treated group during the progress of the experiment at the 10th, 15th, 20th, and 30th week. In a good comparison, LSW-ET treatment, particularly the oral treatment, could dramatically decrease expressions of these factors in mice.

**Figure 4:**
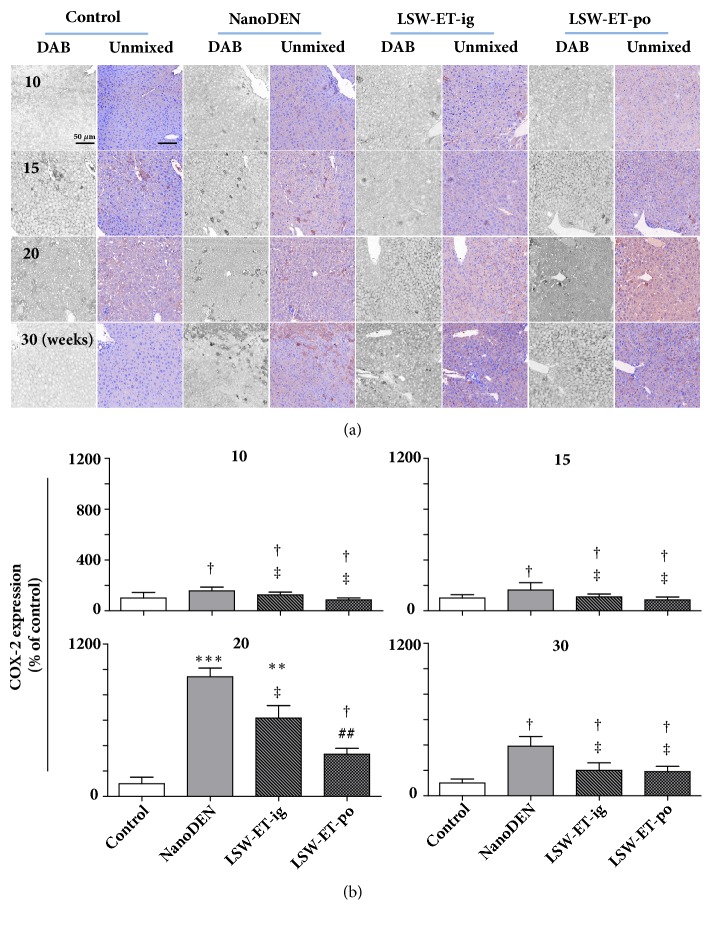
**Oral application of LSW-ET decreased the nanoDEN-induced COX-2 expression in mice. **(a) Expression of COX-2 by immunohistochemistry. Scale bar, 50 *μ*m. (b) Quantitative expression of COX-2 determined by immunohistochemistry. (Results were expressed as the mean ± SEM. *∗*,* P *< 0.05; *∗∗*,* P *< 0.01; *∗∗∗*,* P *< 0.001; †,* P *> 0.01, compared with the control group. #,* P *< 0.05; ##,* P *< 0.01; ###,* P *< 0.001; ‡,* P *> 0.01, respectively, compared with the nanoDEN group.)

In accordance with our previous result that the inflammatory infiltration of mice in nanoDEN group was more severe and LSW-ET treatment could attenuate that, the expression pattern of COX-2 protein levels in liver tissues of nanoDEN group (942 ± 68% and 391 ± 77% of control at the 20th and 30th weeks, respectively) was much stronger than that in LSW-ET-ig group (618 ± 97% and 200 ± 59% of control at the 20th and 30th weeks, respectively). More importantly, the COX-2 expression in the LSW-ET-po group (333 ± 46% and 190 ± 41% of control at the 20th and 30th weeks, respectively) reduced significantly, as shown in Figure 4.

**Figure 5:**
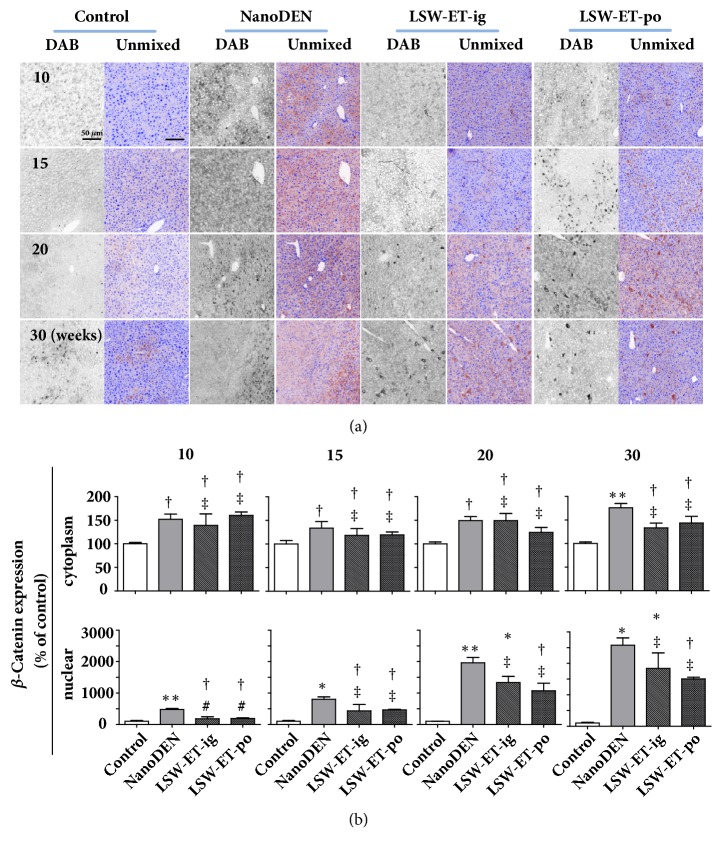
**Oral application of LSW-ET decreased the nanoDEN-induced **β**-catenin expression in mice. **(a) The expression of *β*-catenin by immunohistochemistry. Scale bar, 50 *μ*m. (b) Quantitative expression of the cytoplasmic and nuclear expression of *β*-catenin determined by immunohistochemistry.

Aberrant accumulation of *β*-catenin had been suggested to be quite important during the progression of liver tumor. Therefore, we quantified our liver samples with a multispectrum imaging system to see the difference of nuclear localization of *β*-catenin. As we can see in Figure 5, compared to nanoDEN group (477 ± 32%, 805 ± 72%, 1963 ± 167%, and 2579 ± 232% of control at the 10th, 15th, 20th, and 30th weeks, respectively), the protein level of nuclear *β*-catenin obviously decreased in liver tissue after LSW-ET oral treatment (192 ± 20%, 466 ± 20%, 1075 ± 240%, and 1506 ± 54% at the 10th, 15th, 20th, and 30th weeks, respectively). Similar result was observed in the expression pattern of COX-2 in LSW-ET-ig group (1334 ± 198% and 1838 ± 495% of control at the 20th and 30th weeks, respectively).

**Figure 6:**
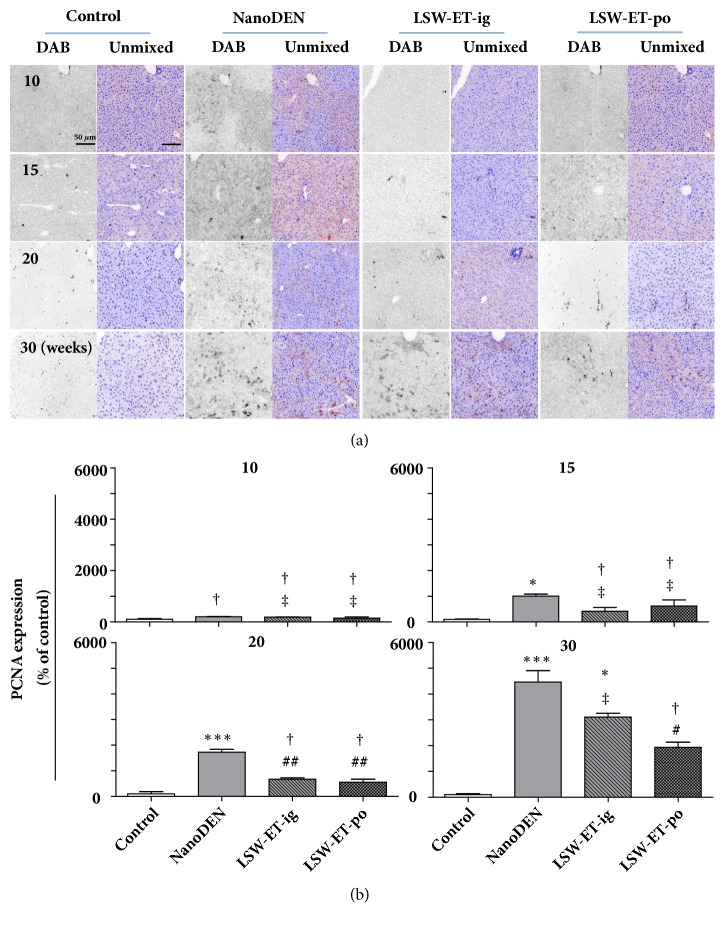
**Oral application of LSW-ET decreased the nanoDEN-induced PCNA expression in mice. **(a) Expression of PCNA by immunohistochemistry. Scale bar, 50 *μ*m. (b) Quantitative expression of PCNA determined by immunohistochemistry.

**Figure 7:**
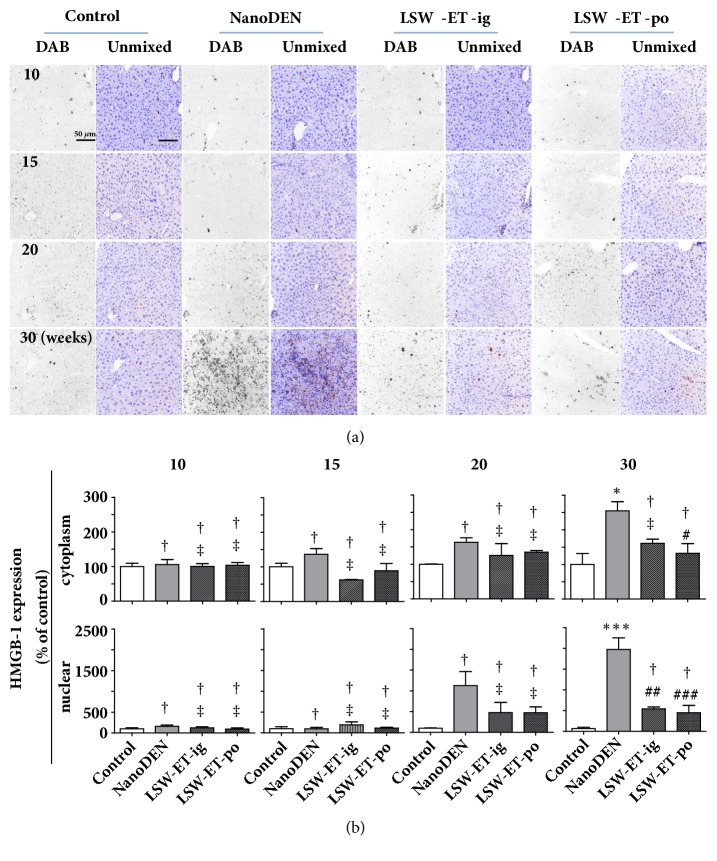
**Oral application of LSW-ET decreased the nanoDEN-induced HMGB-1 expression in mice. **(a) Expression of HMGB-1 by immunohistochemistry. Scale bar, 50 *μ*m. (b) Quantitative expression of the cytoplasmic and nuclear expression of HMGB-1 determined by immunohistochemistry.

Consistently, oral LSW-ET treatment induced decreases in protein levels of PCNA (560 ± 113% and 1937 ± 194% of control at the 20th and 30th weeks, respectively) and HMGB-1 (nuclear: 449 ± 178% of control; cytoplasm: 132 ± 28% of control, at 30th week, respectively) in mice in comparison to that in the nanoDEN group (Figures 6 and 7).

It is also interesting to notice that the decrease of COX-2, *β*-catenin, PCNA, and HMGB-1 expression in the LSW-ET-po group was lower than in the LSW-ET-ig group at week 30, further demonstrating a better antitumor effect of LSW-ET oral application.

## 4. Discussion

According to a recent investigation, hepatocellular carcinoma (HCC), which accounted for approximately 90% of primary liver cancers, became the second most common cause of cancer-related death with an average annual occurrence of about 850 K new cases worldwide [30]. Sorafenib was approved as a first-line systemic therapy for advanced HCC with associated extrahepatic spread and/or vascular invasion in 2007. However, there were several limitations associated with sorafenib therapy. And it extended the survival of advanced HCC patients by only approximately three months compared with placebo [31, 32]. These data underscored the urgent need to develop more effective therapies against liver cancer. Recently, many potential new compounds have been demonstrated to possess anticancer cell abilities, including ailanthone [33], botulin [34], and matrine [35]. However, in vivo experiments were still needed for better illustration of their efficacies.

Here, for the first time, we demonstrated that the LSW-ET, the alcoholic extract of Liushenwan, an herbal preparation in the Traditional Chinese Medicine, could reduce the occurrence and relieve the phenotype of nanoDEN-induced liver cancer in mice. Two different administration methods were used in this study for the delivery of LSW-ET. Results showed that oral administration might be better to reduce the inflammation of livers since it was more efficient in alleviating upregulation of pivotal factors. It is not surprising because most components of LSW-ET (other than 5-HTQ and 5-HT), as can be seen in LC-MS, were of low polarities and hence would be easier to be intake in mouth. This fits well with the traditional usage of Liushenwan, since it was recommended to be taken sublingually.

We previously demonstrated that COX-2, *β*-catenin, and PCNA were upregulated in the liver of tumorous mice induced by nanoDEN. Here, we showed that after the uptake of LSW-ET, these three factors were all recovered (at least partly) compared with the nanoDEN-treated model group. Moreover, elevation of nuclear *β*-catenin in the model group was largely inhibited especially after the oral uptake of LSW-ET. We also examined the expression level of HMGB-1, the expression of which had been demonstrated to control liver cancer initiation via YAP-HIF1*α* pathway. Results showed that, particularly in week 30, when tumors were observed, the nuclear and cytoplasmic expressions of HMGB-1 in LSW-ET-treated groups were greatly reduced compared with the model group, which further strengthened the notion that LSW-ET was effective in preventing the initiation of liver cancer. It is also interesting to note that the inflammation scores of livers were reduced in LSW-ET-po group, which is in accordance with the COX-2 expression levels in liver specimens, suggesting that LSW-ET was not only effective in preventing tumor but also capable of alleviating inflammations in liver. This is not surprised though, since the components of LSW-ET, as demonstrated via LC-MS, possessed anti-inflammatory effect. Nevertheless, this could potentially be insightful since the occurrence of hepatitis, whether virus or alcohol-induced, was not rare, and LSW-ET may contribute to the cure of them.

Another interesting aspect of this study is that, as shown in Table 2, plasmatic LDL-C concentration of the model group is significantly reduced compared with the control group, which is in accordance with the observation that the hair of mice in the nanoDEN group appeared to be dull and yellowish, suggesting an imbalance of lipid metabolism in cancerous mice induced by nanoDEN. In addition, LSW-ET treatment totally reversed the reduction of plasmatic LDL-C concentration indicating that LSW-ET is potentially capable of maintaining the balance of lipid metabolism. More investigations could be conducted to analyze the phenomena and illustrated the possible detailed mechanism.

## 5. Conclusion

In this study, we observed antitumor activity of LSW-ET in nanoDEN-induced HCC in mice. Pathology analysis indicated that modulation on inflammatory-, proliferation-, apoptosis-, and migration-related proteins may contribute together to the enhanced activity of LSW-ET. Our studies suggest that orally uptake of LSW-ET might be a promising prevention and treatment for liver cancer.

## Figures and Tables

**Table 1:** 

**No**	**Ret Time** **min**	**Area MAU** **∗** **min**	**Mass (m/z)**		**Compound**
			**Measure**	**Calculated**	
1	5.170	13.064	219.1490	219.1502	5-HTQ
2	6.033	21.191	177.1026	177.1033	5-HT *∗*
3	26.913	5.752	417.2270	417.2282	Ψ-Bufarenogin
4	27.977	5.755	403.2476	403.2489	Gamabufotali
5	29.733	6.611	217.2270	417.2282	Bufarenogin
6	31.343	9.517	417.2268	417.2282	Arenobufagin
7	31.540	8.257	417.2268	417.2282	Desacetylcinobufotalin*∗*
8	33.757	9.192	403.2476	403.2489	Telocinobufagin *∗*
9	34.130	4.021	401.2322	401.2322	Desacetylcinobufagin
10	34.400	12.225	445.2581	445.2584	Bufotaline *∗*
11	35.190	12.599	459.2370	459.2377	Cinobufotalin *∗*
12	37.187	17.828	387.2524	387.2529	Bufalin *∗*
13	40.767	28.922	443.2421	443.2428	Cinobufagin *∗*
14	41.437	28.623	385.2368	385.2372	Resibufogenin*∗*

*∗*Identities of these peaks were additionally verified with correspondent reference substances.

**Table 2:** Results of the serum chemistry profile analysis in the week 30 mice. The changes in serum ALT, AST, TBIL, and ALP levels directly or indirectly reflect the damage, and the changes in serum TRIG and LDL-C levels reflect the lipid accumulation and metabolism of livers in groups under different treatments. Data were presented as mean ± SEM. Comparing with the control group, *∗* and *∗∗* represent *P <*0.05 and 0.01, respectively, while comparing with the nanoDEN group, # represents *P < *0.05.

	**ALT**	**AST**	**TBIL**	**ALP**	**TRIG**	**LDL-C**
	**(U/L)**	**(U/L)**	**(**μ**mol/L)**	**(U/L)**	**(mmol/L)**	**(mmol/L)**
**Control**	23 ± 3	75 ± 6	8.2 ± 1.1	30.9 ± 2.5	1.52 ± 0.12	1.86 ± 0.14
**nanoDEN**	36.7 ± 2.1**∗**	117 ± 25	11.0 ± 1.1	84 ± 11**∗∗**	1.33 ± 0.07	1.12 ± 0.07**∗**
**LSW-ET-ig**	33 ± 3	111 ± 20	8.9 ± 1.2	48 ± 7**#**	1.34 ± 0.08	1.99 ± 0.24**#**
**LSW-ET-po**	26.6 ± 2.0	91 ± 7	11.6 ± 2.7	62 ± 14	1.77 ± 0.23	2.03 ± 0.10**#**

## Data Availability

The data used to support the findings of this study are available from the corresponding author upon request.
